# Contingency learning of social cues: neural engagement and emotional modulation by facial expressions

**DOI:** 10.3389/fnhum.2025.1527081

**Published:** 2025-02-21

**Authors:** Rahmi Saylik, Burcu Uysal, Adrian Loyd Williams, Robin A. Murphy

**Affiliations:** ^1^Department of Psychology, Mus Alparslan University, Mus, Türkiye; ^2^Department of Psychology, Ibn Haldun University, Istanbul, Türkiye; ^3^Department of Life Sciences, College of Health, Medicine and Life Sciences, Centre for Cognitive & Clinical Neuroscience, Brunel University London, Uxbridge, United Kingdom; ^4^Department of Experimental Psychology, University of Oxford, Oxford, United Kingdom

**Keywords:** contingency learning, emotional stimuli, happy faces, sad faces, attention, fMRI, facial expressions, uncertainty

## Abstract

Contingency learning—the fundamental process by which associations are formed between events in our experience is as relevant of conditioning as it is for social interactions, where emotional cues, such as facial expressions, signal complex and reciprocal causal dynamics. This study investigates the functional neuroanatomy underlying contingency perception by with three type of contingent relation (positive, zero, and negative) using sad and happy facial expressions as stimuli in a group of neurotypical participants. Employing a streaming trial paradigm and functional MRI, we examined how these emotional contingencies engage brain regions involved in attention and predictive processing. The behavioural results indicated that participants could distinguish between different contingencies, regardless of the emotional stimuli. However, judgment ratings varied across conditions, with sad expressions eliciting weaker ratings compared to happy expressions, which moderated perceived causality, especially in the uncorrelated and negative contingency tasks. These behavioural findings were primarily linked to increased activation in frontal regions, including the inferior frontal gyrus, middle frontal gyrus, and anterior cingulate cortex. The results highlight the differential cognitive demands and neural responses evoked by emotional expressions and suggestive of the idea that statistical relations that violate social expectations are processed differently than positive relations.

## Introduction

Contingency learning involves being sensitive to the statistical relationship between cues and outcomes, which is crucial for decision-making, categorization, and causal reasoning (Baker et al., 1996; Heisz et al., 2011). While much research focuses on neutral stimuli, emotional contingencies are essential for maintaining social understanding and navigating interpersonal dynamics, and where responses often interact with others’ emotional states to moderate social relationships (Thornton and Tamir, 2017). For example, interpreting facial expressions predicts others’ emotions and evokes corresponding responses, reinforcing social interactions. This reciprocal exchange, such as a smile prompting a smile, influences both individuals’ emotional states, fostering social cohesion and mitigating potential discord. Understanding and responding to these emotional cues is fundamental to successful social interactions.

Unlike typical emotion recognition tasks, real-world facial expressions are context-dependent and influenced by reciprocal cues between communicators and emotional expressions (Kunar et al., 2014). Rather than remaining in a constant emotional expression, as in emotion recognition tasks, facial expressions in real-world interactions change and are influenced by reciprocal cues between the sender and receiver, which establish contingent events during social interaction (Pavlova and Sokolov, 2022; Straulino et al., 2023). In such situations, emotional valence and social impact can shape the perception of causality based on pre-existing, often egocentric, mental models of emotional transitions (Thornton and Tamir, 2017; Anderson et al., 2019; Barrick et al., 2024). These models may bias perception of causality, particularly in social relational contexts, such as transitions from sadness to neutrality. This is influenced by both subjective and universal affective patterns, shedding light on how emotions may shape learning in dynamic situations.

To test people’s ability to learn these relationships based on statistical reliability, we presented pairs of faces, as if a transmitter and receiver were engaged in a conversation and manipulated four types of events: (a) when a specific emotion was present in both the transmitter (C) and receiver (O), (b) when the emotion was present in the transmitter (C) but absent in the receiver (~O), (c) when the emotion was absent (neutral) in the transmitter (~C) but present in the receiver (O), and (d) when both transmitter and receiver emotions were neutral (~C and ~O). Allan (1980) introduced the Delta P (∆P) rule, a metric for calculating contingency between these events using a 2×2 contingency matrix (Table 1). Delta P (∆P) is computed as the difference between the conditional probability of the outcome given the presence of the cue, P(O|C), and the conditional probability of the outcome given the absence of the cue, P(O| ~ C). The value of ∆P ranges from −1 to +1, with positive values indicating a positive contingency (increasing as events a and/or d increase) and negative values indicating a negative contingency (increasing as events b and/or c increase) (Allan, 1980; Crump et al., 2007; Hannah et al., 2009; Murphy et al., 2022).

**Table 1:** Cue-outcome combinations in a 2 × 2 contingency matrix.

	**O**	**~O**	
**C**	*a*	*b*	$\Delta P=\frac{a}{a+b}-\frac{c}{a+d}$
**~C**	*c*	*d*	

C and ~C represent the presence and absence of the predictive cue (facial expression in this experiment), respectively, and O and ~O represent the presence and absence of the outcome (facial expression), respectively.

The learning of cue-outcome contingencies more generally seems to involve a diverse set of frontal–parietal and temporal regions, such as inferior frontal gyrus (IFG, BA 44/45), middle frontal gyrus (MFG, BA9), superior frontal gyrus (SFG, BA 10), anterior and posterior cingulate gyrus (AC/PC, BA 24-32-23), inferior parietal lobule (IPL, BA 40) and superior temporal gyrus (STG BA 43) (Fletcher et al., 2001; Tsukiura et al., 2003; Turner et al., 2004; Carter et al., 2006; Tanaka et al., 2008; Klucken et al., 2009; Mullette-Gillman and Huettel, 2009; Liljeholm et al., 2011; Spiers et al., 2017; Morris et al., 2022). For instance, Turner et al. (2004) investigated the role of prefrontal cortices in an associative learning task in which participants were required make judgments about how certain foods (cues) were related to an allergic reaction (outcome) based on being exposed to multiple trials with the same categories of events involved in Delta P. The study showed that contingency judgments involved positive activations in lateral prefrontal regions (e.g., IFG and MFG). Liljeholm et al. (2013) found that contingency learning was associated with significant brain activations in prefrontal regions, including the lateral and medial prefrontal cortex (medPFC) and IFG. It has been suggested that these regions are part of a goal directed system that may rely on computing relative values of cues as possible causal candidates (Turner et al., 2004; Tanaka et al., 2008; Liljeholm et al., 2013).

Evidence from contingency judgment studies further refines this understanding by demonstrating that attentional resource allocation varies with contingency type (Maldonado et al., 2006; Saylik et al., 2022). First, zero-contingency tasks, characterized by the absence of predictive relationships between cue and outcome, represent a form of uncertainty that may elicit stress and demand significant cognitive and neural resources (Behrens et al., 2007; Le Pelley et al., 2010; Murphy et al., 2011; Matute et al., 2015; Peters et al., 2017; Stolyarova et al., 2019). The dorsomedial prefrontal cortex (DMPFC), lateral prefrontal cortex (LPFC), and temporal regions are critical for identifying shifts in environmental contingencies and updating cognitive models to accommodate the absence of clear patterns and formation of causal biases (Mullette-Gillman and Huettel, 2009; Spiers et al., 2017). Complementing these regions, the anterior cingulate cortex (ACC) plays a pivotal role in managing uncertainty by integrating volatility signals and modulating learning rates, enabling individuals to adaptively respond to uncorrelated outcomes (Behrens et al., 2007). Second, negative contingencies are also considered demanding because it is more difficult when the presence of a cue signals the absence of an outcome (Maldonado et al., 2006; Saylik et al., 2022). For instance, Heisz et al. (2011) employed a streaming paradigm with emoticons, revealing that negative contingencies elicit stronger activity in attention-related brain areas compared to positive contingencies. Extending this, our functional magnetic resonance imaging (FMRI) study used geometric shapes (triangles and hexagons) to examine contingency judgments. We observed that negative contingencies led to higher activation in areas such as the IFG and MFG, whereas a common neural response was found in the posterior cingulate cortex (PCC), supramarginal gyrus, and STG across all contingencies (Saylik et al., 2022). Finally, positive contingencies may involve more automatic processes, as it is easier to follow co-presence and co-absence of cues and outcomes, and thus elicit less activation in cognitive control areas (Maldonado et al., 2006; Saylik et al., 2022). Taken together, zero and negative contingencies demand greater cognitive and neural resources due to their inherent uncertainty and complexity, engaging regions like the DMPFC, LPFC, and ACC compared with positive contingencies.

In addition, emotions interact with higher-level cognitive processes, including decision-making and judgment, by altering how individuals perceive, interpret, and evaluate available information (Blanchette and Richards, 2010; Brosch et al., 2013). The effect of emotional stimuli may become more pronounced in uncertain and demanding conditions (e.g., zero or negative contingencies) due to the increased levels of uncertainty and cognitive difficulty involved. Two potential mechanisms may explain these effects: the availability heuristic and the narrowed attention hypothesis. The availability heuristic suggests that emotions influence judgments by affecting memory retrieval, with mood-congruent memories being more accessible (Blanchette and Richards, 2010). The attention-narrowing hypothesis posits that negative emotions may elevate arousal levels, leading individuals to focus on certain aspects of information, such as saliency, during decision-making and judgment (Easterbrook, 1959; Blanchette and Richards, 2010; Wichary et al., 2016). In this regard, sad emotional stimuli may lead to more conservative and critical judgments by priming sadness related negative exemplars through the availability heuristic or by narrowing attention due to elevated arousal associated with the uncertainty and stress inherent in sadness (Tiedens and Linton, 2001; Blanchette and Richards, 2010; Wichary et al., 2016; Anderson et al., 2019). Happy emotional stimuli, on the other hand, may enhance the retrieval of positive associations, promoting more optimistic and liberal judgments by priming positive exemplars through the availability heuristic and broadening the scope of attention, as they do not elicit stress-related arousal levels (Blanchette and Richards, 2010; Evans et al., 2010). Together, these mechanisms demonstrate how emotions dynamically modulate cognitive strategies, influencing perception and judgment based on the emotional context (Anderson et al., 2019).

The effect of negative emotional stimuli on cognitive processes appears to depend on task demands and the balance between bottom-up emotional processing and top-down cognitive control. One line of research suggests that negative emotions enhance memory-guided attention by increasing activity in frontoparietal, insular, limbic and parahippocampal regions, indicating aheightened allocation of attentional resources to task-relevant stimuli in visual search tasks (Brosch et al., 2013; Pedale et al., 2019; Salsano et al., 2024). In this context, the interference from negative emotional stimuli acting as distractors can be resolved through enhanced connectivity between the insular cortex and prefrontal regions, along with increased heartbeat-evoked responses, which have been shown to be modulated by emotional arousal (Dolcos and McCarthy, 2006; Anticevic et al., 2010; Kim et al., 2019; Pedale et al., 2019). However, this cognitive effort, along with increased fronto-parietal activity, may be accompanied with more conservative judgments and reasoning in the conditions with negative emotional stimuli (e.g., sad faces) compared to those with positive emotional stimuli, which tend to facilitate cognitive control processing (Calvo and Nummenmaa, 2008; Yuan et al., 2023). While sad stimuli weaken causal perception and reduce valence ratings in contingency judgments, leading to more conservative judgments (Young et al., 2020), happy faces offer an advantage in tasks like visual search (Calvo and Nummenmaa, 2008) and associative learning (Evans et al., 2010), including probabilistic tasks where they enhance reward processing and motivation, encouraging more exploratory behaviour, even when no clear contingency exists (Averbeck and Duchaine, 2009; Saylik et al., 2021). The processing of happy faces may enhance contingency judgment processes and reduce interference in frontoparietal activation, while negative emotional stimuli often disrupt attention allocation, leading to stronger activations (Dolcos and McCarthy, 2006; Anticevic et al., 2010; Pedale et al., 2019). As the processing of zero and negative contingencies requires more attentional resources, this effect could be more pronounced in such conditions. Taken together, these findings suggest that emotion-related brain regions interact with traditional associative learning networks, with emotional valence modulating attention allocation and contingency evaluations (Furl et al., 2012; Keskin-Gokcelli et al., 2024; Wang et al., 2024).

The current study aims to extend the investigation of contingency learning by examining how different types of contingencies (negative, zero, and positive) are processed when associated with happy and sad facial expressions. Utilizing a streaming trial procedure adapted from Crump et al. (2007) and employing functional magnetic resonance imaging (fMRI), we investigate the neural correlates underlying the processing of these emotional contingencies. In our study, we use two distinct types of stimuli to evoke emotional responses and explore their effects on learning processes. Negative stimuli are represented by sad facial expressions, which may be associated with uncertainty and increased arousal levels (Tiedens and Linton, 2001; Anderson et al., 2019). In contrast, positive stimuli are represented by happy facial expressions, which may be linked to certainty and approachability (Tiedens and Linton, 2001). Emotional expressions appear to affect judgment, reasoning and decision-making process due to various factors, including mood, psychological disorders, pre-existing mental models of emotional transitions leading biases, as well as valence of emotions (Blanchette and Richards, 2010; Brosch et al., 2013; Thornton and Tamir, 2017; Matute et al., 2019; Barrick et al., 2024). These happy and sad stimuli are integrated with three types of contingency relations (i.e., positive, zero, negative) to examine the functional neuroanatomical correlates of contingency judgment in neurotypical participants. We hypothesize that contingencies involving sad facial stimuli will lead to a weaker perception of causality and will more robustly engage frontal brain regions in negative and zero contingency tasks compared to positive contingencies. This reflects the increased cognitive demands required for processing negative and zero contingencies during the judgment process.

## Methods

### Participants and materials

Twenty-nine healthy participants (14 females, 15 males), aged between 18 and 28 years (males: M = 23.30, SD = 3.60; females: M = 22.40, SD = 3.29) participated in the study. Each participant provided written informed consent and received £20 for their one-hour participation. Our sample size was based on similar studies within the field (Morris et al., 2022; Saylik et al., 2022). The study was approved by the Department of Life Sciences Research Ethics Committee at Brunel University London.

To control for potential confounding factors in contingency learning, participants completed a series of questionnaires prior to the experiment. Participants were university students fluent in English, scored below 20 on Beck’s Depression Inventory (BDI; M = 12.33, SD = 3.94) (Beck et al., 1988), and had no reported history of psychiatric or neurological disorders. Intelligence Quotient (IQ) was approximated using demographic characteristics (Crawford and Allan, 1997; M = 112.17, SD = 5.54), aligning closely with the National Adult Reading Test (Nelson and Willison, 1991).

### Contingency learning task

The contingency learning task involved the rapid presentation of cue and outcome pairs, following the procedures described by Crump et al. (2007), and closely matched the task used in a previous study (Saylik et al., 2022), with the inclusion of facial emotional stimuli. Stimulus delivery and response collection were facilitated by PsychoPy3 (Peirce et al., 2019).

**Figure 1:**
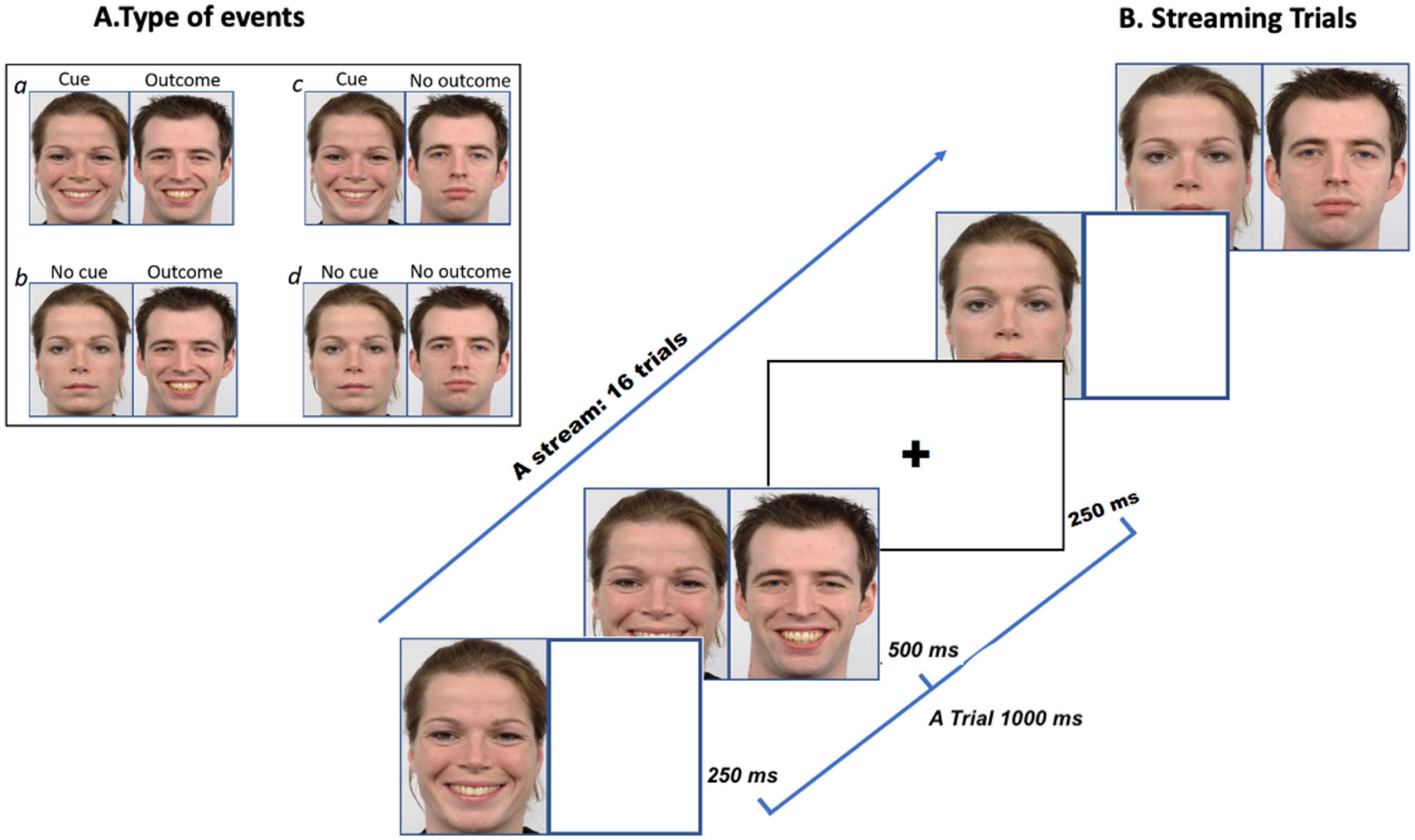
Panel **A** illustrates an example of four possible events a, b, c, d. Panel **B** illustrates the rapid presentation of the cue and outcome in a happy face condition (Reproduced from Langner et al., 2010).

We selected images of 10 individuals (5 females and 5 males) from the Radboud Faces Database (RaFD; Langner et al., 2010), each measuring 681×1,024 pixels. The RaFD images are standardized for salience (Langner et al., 2010), with physical matching and control over factors such as facial landmarks, lighting, and background. This widely utilized database (Jaeger, 2018) achieves a recognition accuracy of 88% across various cultures (Mishra et al., 2018). Each face displayed three expressions—neutral, sad, and happy—resulting in a total of 30 facial images. During the behavioural practice session, we used images of two individuals showing neutral, sad, and happy expressions. In the main experiment, images of eight individuals displaying the same emotional expressions were used. In each contingency condition, two faces were used. The gender of each photo, nominally assigned by RaFD, was not explicitly labelled. The gender of the face was randomized across trials as either Person A or Person B. As shown in Figure 1, the cue was the initial face and expression, while the outcome reflected either the same emotion as the initial face or a neutral expression. Faces were categorized into two types of events: emotional (+C and +O events) or neutral (~C and ~O events) as shown in Figure 1. The events were as follows:

**a.** Person A’s emotional expression predicts that Person B will express the same emotion (e.g., both are happy, indicating that both the cue and outcome are present; +C, +O).
**b.** Person A’s emotional expression predicts that the absence of emotion in Person B (e.g., Person A is happy, while Person B remains neutral, indicating that the cue is present, but the outcome is absent; +C, ~O).
**c.** The absence of emotional expression in Person A predicts the presence of emotion in Person B (e.g., Person A is neutral, while Person B is happy, indicating that the cue is absent, but the outcome is present; ~C, +O).
**d.** The absence of emotional expression in Person A predicts the absence of emotion in Person B (e.g., both Person A and Person B are neutral, indicating that both the cue and the outcome are absent; ~C, ~O).

Delta P (ΔP) was manipulated to create three levels of contingency: negative (ΔP = −0.50), zero (ΔP = 0), and positive (ΔP = 0.50) for both happy and sad faces (Table 2). The study involved seven conditions: three emotive tasks (negative, zero, and positive contingencies) for each emotion (happy and sad), plus one baseline condition that included a black fixation cross in the center of white screen. Each task condition was presented 8 times in a randomized order.

**Table 2:** Illustrates the distribution of trials for each cell ‘a= Cue (+C) and Outcome (+O), b= C+ and No Outcome (–O), c= No Cue (–C), and +O, d= –C and –O along with statistical relations for conditional probability (P of O) and Delta P (ΔP).

Negative				Zero				Positive			
	+O	–O	P		+O	–O	P		+O	–O	P
+C	2	6	0.25	+C	4	4	0.5	+C	6	2	0.75
–C	6	2	0.75	–C	4	4	0.5	–C	2	6	0.25
Sum of +O	8		𝛥P	Sum of +O	8		𝛥P	Sum of +O	8		𝛥P
			−0.5				0				0.5

Each task condition formed a block comprising a stream of 16 trials with a behavioural judgment at the end. Within the blocks, each 1,000 ms trial began with a fixation cross displayed for 250 ms, followed by a cue on the left side of the screen for 250 ms, and then an outcome on the right side of the screen for 500 ms, all presented on a white background. A new trial began after another fixation cross presentation. At the end of the stream of 16 trials, participants rated their predictions on a scale ranging from −4 to +4. After making judgments or 5 s passed without response, the next block began, with instructions for the upcoming block presented for a fixed 5 s. Each block was repeated 8 times, ensuring that each condition accumulates a total of 168 s of functional data (i.e., trial presentation plus behavioural judgement).

### Procedure

Participants first completed a behavioural practice session involving positive, zero, and negative contingencies with using sad and happy facial stimuli. The stimuli in the practice session, consisted of images of male and female faces that were not included in the main experiment. The practice session lasted approximately 7 min. During the main experiment, participants lay supine in an MRI scanner, holding two MRI-compatible response pads, wearing MRI-compatible in-ear headphones, and viewing a 1,024×768 screen at a distance of 85 cm via a mirror system. Conditions were presented for 16 s within an fMRI blocked design. Following each condition, participants viewed a response scale for up to 5 s, with ratings from −4 to 0 on the left-hand keypad and +1 to +4 on the right-hand keypad.

Similar to a social conversation, the emotional state of one person (the transmitter) can trigger a reaction in the other person (the receiver), or it may have no effect. This implies that Person B’s expression could have been caused by Person A’s expression. On this basis, participants were instructed to imagine two people having a conversation while carefully observing the stream of cue and outcome pairs. At the end of the presentation, participants were asked to judge the relationship between the facial expressions of Person A and Person B—specifically, whether Person A’s emotion predicted the presence or absence of an emotion in Person B based on the presented scale on the screen.

### MRI procedure

Imaging was conducted using a 3 T scanner (Trio, Siemens, Erlangen, Germany) equipped with a 12-channel array head coil. Participants lay flat on the scanner bed with head motion minimized using cushions. Functional images were acquired using a BOLD-sensitive gradient echo EPI sequence (TR = 2.5 s, TE = 31 ms, 85° flip angle), with 35 axial slices (192 × 192 mm FOV, 64 × 64 matrix, 3 × 3 mm in-plane resolution, 3 mm thickness, no gap, interleaved slice acquisition), and 760 volume acquisitions. High-resolution anatomical images were obtained using a T1-weighted MPRAGE sequence (TR = 1,900 ms, TE = 3.03 ms, 11° flip angle, 176 slices, 256 × 256 mm FOV, 1 × 1 × 1 mm voxel size). Each participant completed one functional and one anatomical scan.

### Data analysis

MRI data analysis was performed using SPM 12. Initially, the structural and functional images were manually aligned with the anterior commissure, followed by head motion correction (Realign & Unwarp). Images were normalized to MNI space using unified segmentation, preserving the acquisition resolution of 3x3x3 mm; this was followed by spatial smoothing with an 8 mm FWHM Gaussian kernel. Normalization and registration success were validated through visual inspection.

Statistical analysis employed a general linear model for serially autocorrelated observations, based on voxel-wise least-squares estimation (Friston et al., 1994). The BOLD response was modelled with a boxcar function convolved with a canonical HRF without derivatives. A cut-off frequency of 1/128 Hz was used for the high-pass filter. Boxcar duration was variable, reflecting the variable response times and block lengths, but was typically around 17–21 s. First-level statistics calculated all contrasts of interest per participant, with second-level analysis using one-sample t-tests. Significance was thresholded at *p* < 0.05 (FWE corrected) and also at *p* < 0.001 (uncorrected) for all t-maps, with significant activations at the cluster level reported at *p* < 0.05 (FWE corrected). Anatomical regions and Brodmann areas were identified using the Automated Anatomical Labelling toolbox (AAL3v1; Tzourio-Mazoyer et al., 2002; Eickhoff et al., 2005).

We first generated an interaction contrast (1 1 1 −1 −1 −1) to examine the effect of sad and happy faces across the three contingencies [Sad face (Negative, Zero, Positive Contingencies) – Happy face (Negative, Zero, Positive Contingencies)]. We also generated contrasts to explore specific differences between contingencies with happy and sad stimuli for each contingency type: negative, zero and positive. Three contrasts, along with their reversed versions, were examined: [_negative contingencies_ (sad stimuli – happy stimuli)], [_zero contingencies_ (sad stimuli – happy stimuli)], and [_positive contingencies_ (sad stimuli – positive) happy stimuli]. This approach aimed to reduce confounding effects from task-irrelevant cortical activations (Szameitat et al., 2011; Saylik et al., 2022).

Research suggests that biases in judgment may appear in early trials and dissipate in later trials (Murphy et al., 2011; Matute et al., 2019). From an exploratory perspective, given that we employed emotional stimuli, we investigated changes in participants’ judgments across the 8 block repetitions by dividing tasks into two halves: the first four repetitions (early trials) and the last four repetitions (late trials). The contrasts for this analysis were the same as those described earlier but were calculated separately for the early or late trials. This resulted in a total of nine contrasts [e.g., _early trials negative contingencies_ (sad stimuli – happy stimuli)], [_late trials zero contingencies_ (sad stimuli – happy stimuli)]. Behavioural responses were averaged and analysed for each condition, with additional analyses conducted separately for early and late trials. Neural responses were then analysed using corresponding regressors.

### Behavioural results

**Figure 2:**
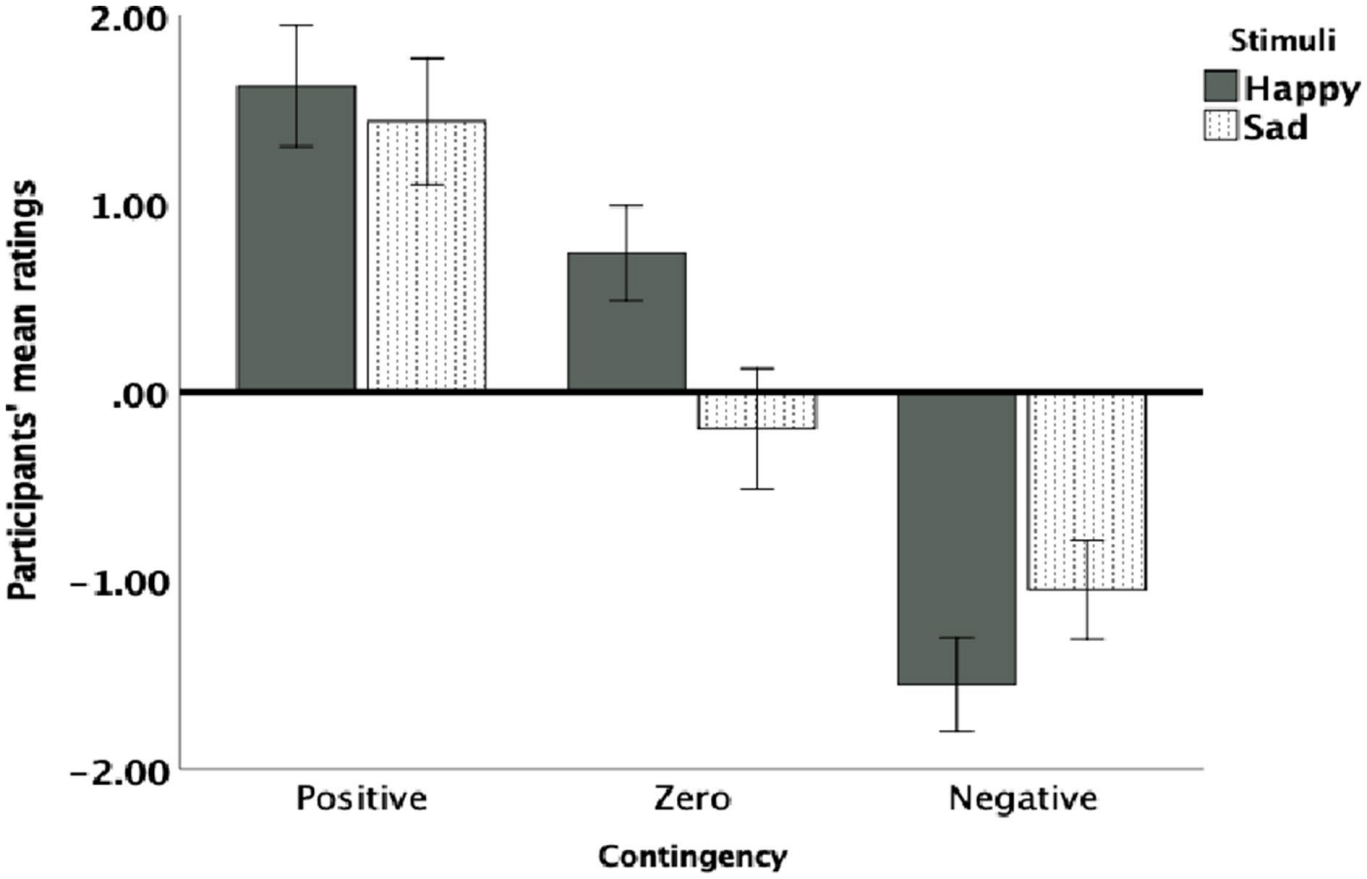
Participants’ mean contingency ratings for each contingency (negative, zero, and positive) and stimulus type (happy and sad). Error bars represent 95% confidence intervals (CI).

As shown in Figure 2, participants demonstrated sensitivity to the three statistical contingencies, with stronger discrimination between contingencies when happy faces were used. As described in the data analysis section, task repetitions were divided into two halves: the first half was labelled “early trials,” and the second half was labelled “late trials.” Both early and late trials were included in the analysis. A 3 × 2 × 2 factorial ANOVA was conducted with the within-subject factors of contingency (negative, zero, positive), trials (early, late), and stimulus type (happy, sad). The analysis revealed a significant main effect of contingency, *F* (2,27) =52.260, *p* < 0.001, *η2* = 0.68, indicating that participants effectively discriminated among negative, zero, and positive contingencies. However, the main effects of stimulus type (*F* (1,29) =0.829, *p* = 0.371) and trials (*F* (1,28) =1.390, *p* = 0.250) were not significant, suggesting that overall ratings were not influenced by stimulus type (happy or sad) or by trial timing (early or late). Likewise, the interaction effect among contingency, stimuli, and trials was not significant (*F* (2,27) =0.275, *p* = 0.602). However, a significant interaction between contingency and stimulus type was observed, *F* (2,27) = 7.341, *p* = 0.002, *η*^2^ = 0.21, indicating that contingency discrimination varied across different stimulus types. Further, contrasts analysis demonstrated significant stimuli effect across early and late trials for zero contingency (*F* (1,28) = 13.726, *p* = 0.001, *η2* = 0.35) and for negative *F* (1,28) = 3.404, *p* = 0.048 but not for positive contingency (*F* (1,28) = 0.29, *p* =  0.551).

*Post hoc* analyses with Bonferroni correction were conducted for significant effects. For the negative contingency task, overall, happy faces were rated as more contingent than sad faces, though this approached statistical significance, (*MD* = 0.755, *SE* = 0.380, 95% *CI*: [−0.044, 1.544], *p* = 0.060). In the early trials of negative contingency, the difference between happy and sad faces were significant (*MD* = 0.500, *SE* = 0.241, 95% *CI*: [−0.12, 0.988], *p* = 0.045) but it was not significant in the late trials (*MD* = 0.263, *SE* = 0.363, 95% *CI*: [0.481, 1.000], *p* = 0.472).

**Figure 3:**
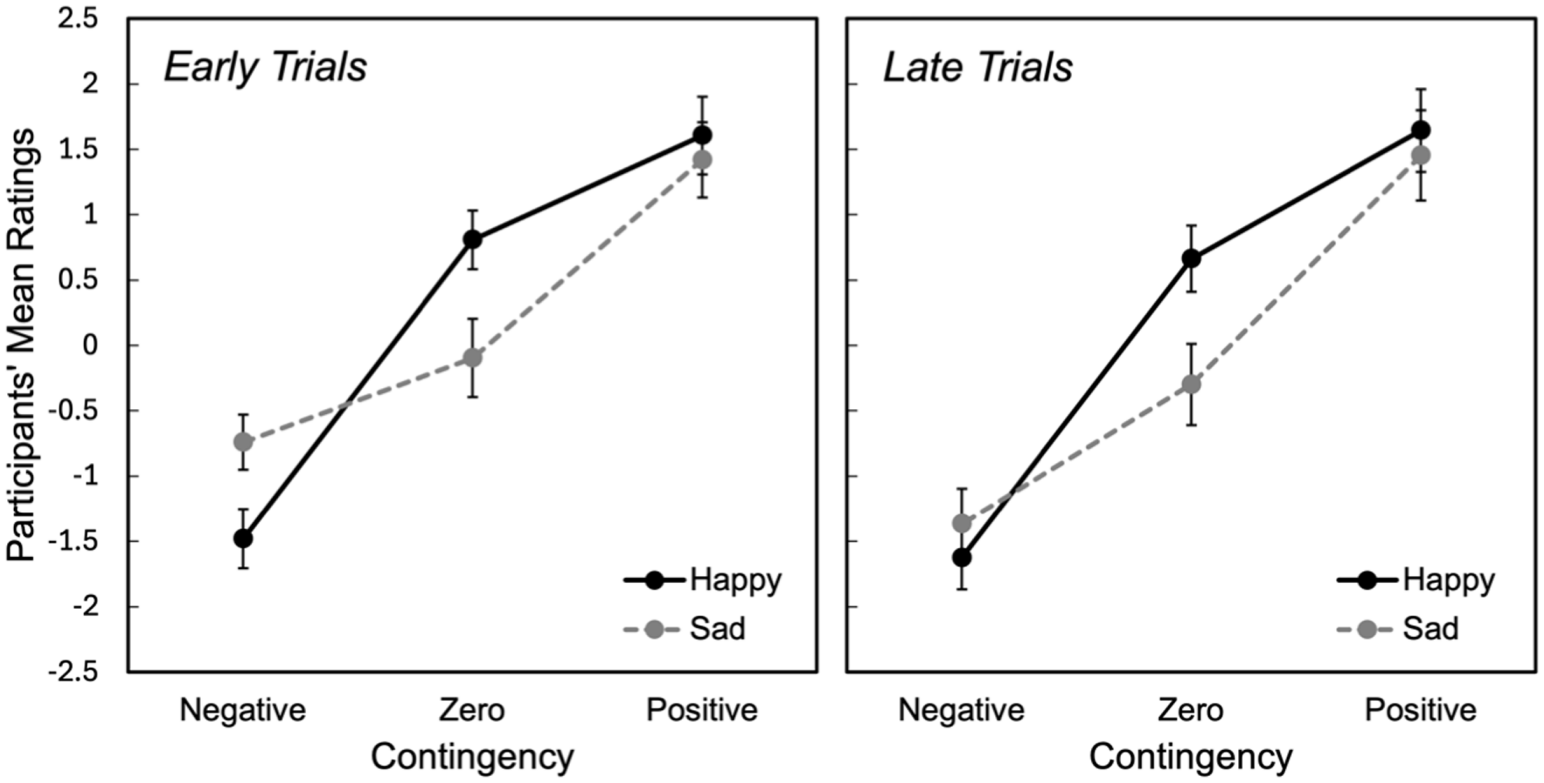
Participants’ mean ratings for the first and the second halves of the trials across negative, zero, and positive contingencies for both happy and sad stimuli. Error bars represent the standard error of the mean (SEM).

For zero contingencies, overall happy faces were rated more positively while sad faces were rated more negatively, with a significant difference between stimuli [*MD* = 0.685, *SE* = 0.334, 95% *CI*: (0.002, 1.335, *p* = 0.012)] (Figure 2). As can be seen in Figure 3, both in the early and late trials of zero contingency, the difference between happy and sad faces were significant [early trials; *MD* = 0.946, *SE* = 0.295, 95% *CI*: (0.36, −1.510), *p* = 0.002; late trials; *MD* = 0.967, *SE* = 0.443, 95% *CI*: (0.62, −1.860), *p* = 0.037]. No significant differences were observed for positive contingencies across early and late trials [*MD* = 0.179, *SE* = 0.19, 95% *CI*: (−0.217, 574), *p* = 0.304].

### Neuroimaging results

**Figure 4:**
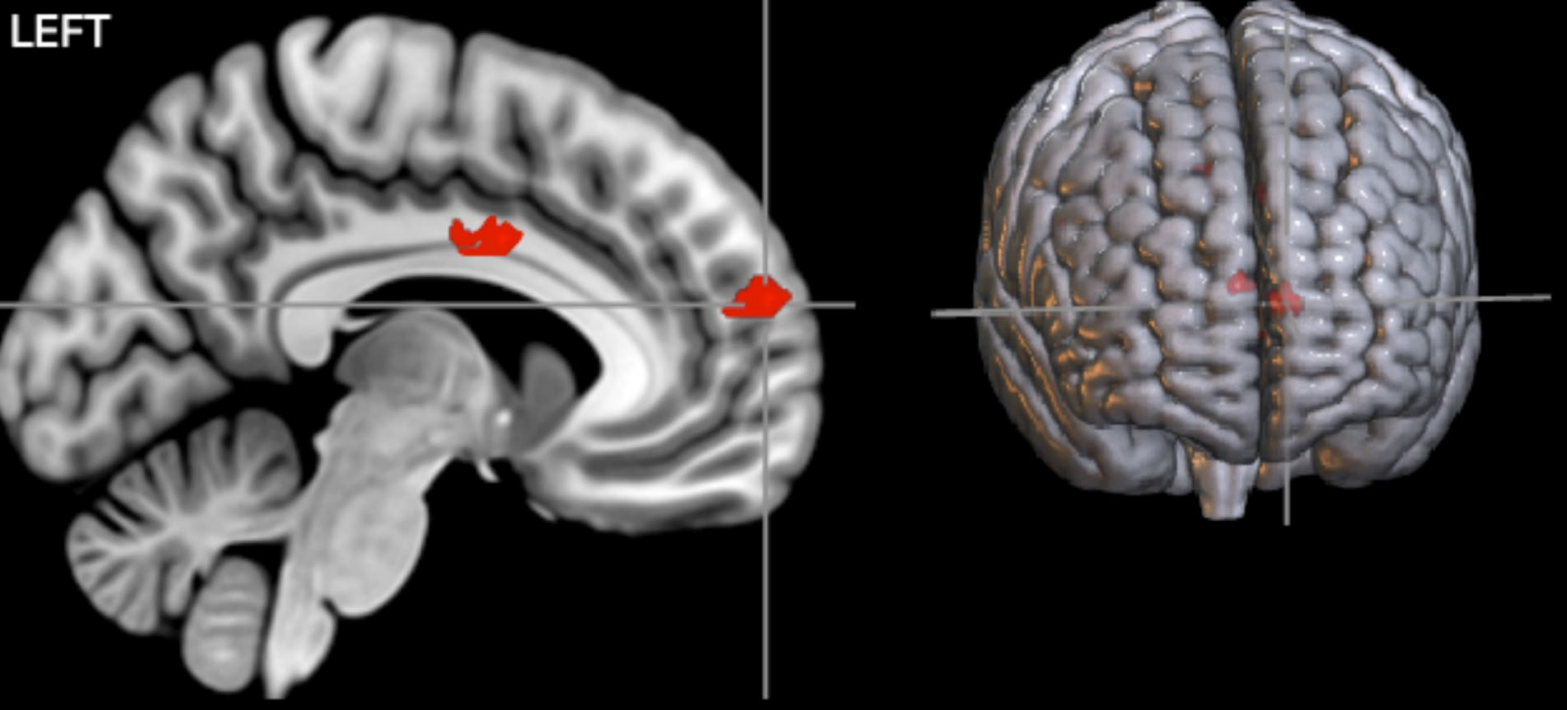
The results of the interaction contrasts. Two significant clusters, primarily covering MedFG and the cingulate cortex, showed significant activations during contingency judgment tasks for sad compared to happy faces. The map was thresholded at a voxel-level *p* < 0.001 (uncorrected) and cluster-level *p* < 0.05 (FWE corrected).

**Figure 5:**
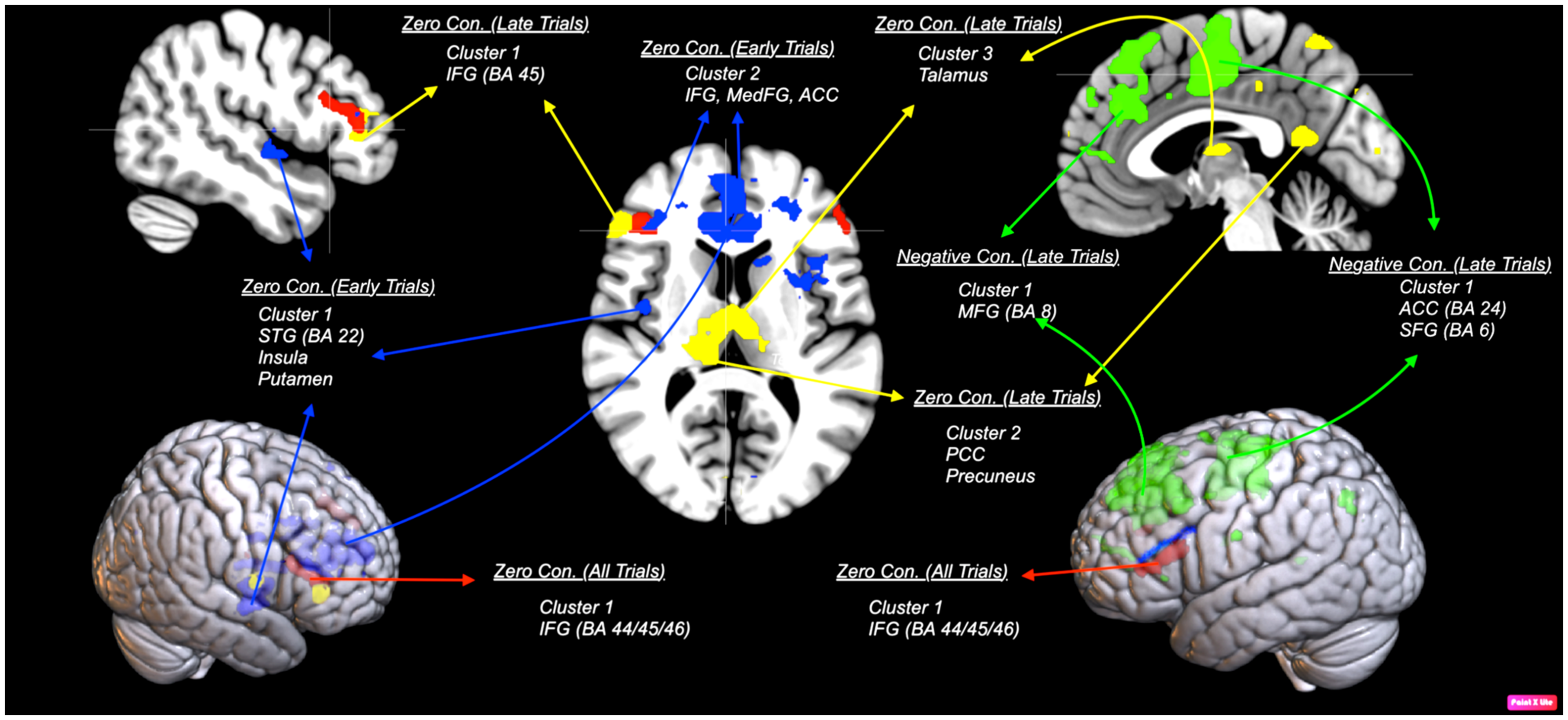
An illustration of areas corresponding to each contrast comparing sad and happy stimuli. Red areas show bilateral activation in the inferior frontal gyrus (IFG; BA 45/44) during zero contingency tasks [_zero contingencies_ (sad stimuli – happy stimuli)].The map was thresholded at voxel-level and cluster-level *p* < 0.05 (FWE corrected). Blue areas show early trials in zero contingency tasks, revealing two clusters with stronger activation: one in the superior temporal gyrus (STG; BA 22), extending to the putamen and insula (BA 13), and another in the inferior (BA 45) and medial frontal gyrus (BA 10), including the anterior cingulate cortex (ACC; BA 24), during the first four trials comparing sad to happy stimuli [_early trials zero contingencies_ (sad stimuli – happy stimuli)]. Green areas show early trials in negative contingency tasks, presenting stronger activation in two clusters: one in the middle frontal gyrus (MFG; BA 8) and another in the ACC (BA 24) and superior frontal gyrus (SFG; BA 6), during the first four trials comparing sad to happy stimuli in negative contingency conditions [_early trials negative contingencies_ (sad stimuli – happy stimuli)]. Yellow areas illustrate late trials in zero contingency tasks, reveal three clusters with stronger activation: one in the IFG (BA 45/44) and ACC (BA 32), another in the posterior cingulate gyrus (BA 23), extending to the precuneus (BA 7), and a third in the thalamus, comparing sad to happy stimuli [_late trials zero contingencies_ (sad stimuli – happy stimuli)]. The map was thresholded at voxel-level *p* < 0.001 (uncorrected) and cluster-level *p* < 0.05 (FWE corrected).

First, we examined an interaction contrast [Emotional Stimuli (Sad vs. Happy) × Contingencies (Negative vs. Zer vs. Positive)]. The results demonstrated contingency judgment lead increased activations mainly in left ACC (BA24) and MedFG (BA 10) for sad compared to happy faces. To further investigate this interaction, we examined contrast comparisons for each type of contingency (Figure 4, Table 3-interaction contrast). As shown in Figure 5 and Table 3, the contrast between zero contingencies with sad versus happy stimuli, [_zero contingencies_ (sad stimuli – happy stimuli)], revealed significant activations in two clusters located in the left and right inferior frontal gyrus (BA 44, 45, 46).

**Table 3:** The table presents the MNI coordinates of significant clusters identified during contingency judgment tasks.

Contrasts	Anatomical area	BA	*x*, *y*, *z*	t/p (uncorr)	Cluster-level *p*(FWE)	Cluster volume (mm^3^)
Overall interaction contrast	Cluster 1			3.83/ 0.025	0.036	120
	ACC	24	−6, −4, 32			
	ACC	24	−12, −28, 44			
	PCC	31	9, −1, 41			
	Cluster 2					
	MedFG	10	−9, 62, 20			232
	MedFG	10	6, 56, 23			
	MedFG	10	0, 53, 8			
All trials						
Zero (Sad – Happy)	Cluster 1			4.51/ 0.001	0.001	2,232
	IFG	45	46, 36, 8			
	IFG	44	44, 18, 20			
	IFG	46	46, 28, 16			
	Cluster 2			6.88/0.010	0.001	1,352
	IFG	44	−48, 20, 20			
	IFG	46	−46, 40 6,			
	IFG	45	−52, 32, 10			
Early trials						
Zero (Sad – Happy)	Cluster 1			4.34/ 0.020	0.045	3,320
	STG	22	50, −8, −6			
	Putamen		32, −2, 2			
	Insula	13	40, −8, 4			
	Cluster 2			4.15/0.001	0.003	7,488
	IFG	45	−30, 32, 2			
	ACC	24	6, 30, 6			
	MedFG	10	6, 54, 10			
Negative (Sad – Happy)	Cluster 1			4.51/ 0.001	0.004	19,120
	MFG	8	−18, 34, 44			
	MFG	8	−28, 22, 48			
	MFG	8	−38, 18, 40			
	Cluster 2			4.14/0.008	0.045	10,184
	ACC	24	−2, −4, 50			
	SFG	6	−28, −24, 68			
	Precent.G	6	−22 -14 68			
Late trials						
Zero (Sad – Happy)	Cluster 1			4.99/ 0.001	0.001	33,064
	IFG	45	50, 34, 4			
	IFG	44	60, −2, 14			
	ACC	32	12, 8, 42			
	Cluster 2			4.41/0.001	0.008	7,488
	PCC	23	−2, −54, 16			
	Precuneus	7	−4, −54, 16			
	Precunes	7	2, −76, 40			
	Cluster 3			4.28/0.006	0.026	
	Thalamus		0, −8, 8			
	Thalamus		10, −28, 12			
	Thalamus		−6, −14, 10			

It includes results for a general contrast: [_negative contingencies_ (sad stimuli – happy stimuli) + _zero contingencies_ (sad stimuli – happy stimuli) + _positive contingencies_ (sad stimuli – happy stimuli)]. The table also includes data for zero contingencies across all trials, as well as early and late trials for judgments of zero and negative contingencies.

In contrast, no significant activations were observed for the contrast [(positive) sad stimuli – (positive) happy stimuli] or [(negative) sad stimuli – (negative) happy stimuli], nor their reversed versions, either at the cluster level (*p* < 0.05, FWE corrected), or even at a more liberal threshold (uncorrected *p* < 0.005).

Further analysis focused on neural responses during early and late trials. For early trials, the contrast for zero contingencies, [_early trials zero contingencies_ (sad stimuli – happy stimuli)] revealed significant activations in two clusters: one covering the right superior temporal gyrus (BA 22), extending into the putamen and insula (BA 13), and the other spanning the left inferior frontal gyrus (BA 45), extending to the right anterior cingulate gyrus (BA 24) and medial frontal gyrus (BA 10). Likewise, for negative contingencies, [_early trials negative contingencies_ (sad stimuli – happy stimuli)], significant activations were found in two clusters within the left frontal areas (uncorrected *p* = 0.005): one in the left middle frontal gyrus (BA 8) and the other covering the anterior cingulate gyrus (BA 24), extending into the superior frontal (BA 6) and precentral gyrus (BA 6). However, the contrast between positive contingencies for sad versus happy stimuli [(positive) sad stimuli – (positive) happy stimuli] did not show significant activations at the cluster level *p* < 0.05 (FWE corrected) or at more liberal thresholds (uncorrected *p* < 0.005).

For late trials, significant activations were observed only in the zero contingency condition comparing sad and happy faces, [_late trials zero contingencies_ (sad stimuli – happy stimuli)]. This comparison revealed three clusters: one in the inferior frontal gyrus (BA 44/45), extending into the anterior cingulate gyrus (BA 32), a second in the posterior cingulate gyrus (BA 23) extending into the precuneus (BA 7), and a third bilaterally covering the thalamus. Comparisons between sad and happy faces for positive [(positive) sad stimuli – (positive) happy stimuli] and negative [(negative) sad stimuli – (negative) happy stimuli] contingencies did not yield significant activations.

## Discussion

We explored functional neuroanatomical correlates of emotional facial expressions (happy and sad) during different contingencies (negative, zero, and positive). Overall, our results revealed specific effects where emotional stimuli modulate the perception of causality, particularly in zero contingency conditions. Behavioural results indicated that participants perceived slightly negative sense of causality when judging zero contingency conditions with sad faces, while they perceived a slightly positive sense of causality happy faces were involved. This pattern of results was consistent across both early and late trials of the zero contingency task. Furthermore, participants perceived a weaker sense of causality in negative contingency conditions with sad faces during early trials, with this effect diminishing in the late trials.

These behavioural findings were accompanied with increased activity in bilateral lateral frontal regions, ACC and additional subcortical areas. In the early trials, sad faces during zero contingencies, compared to happy faces, led to heightened activation in broader lateral frontal regions, including the anterior cingulate and temporal regions (such as the superior temporal gyrus (STG)), which extended into the insula, thalamus, and putamen. These activations continued into the late trials, involving deeper structures like the posterior cingulate cortex (PCC), thalamus, and precuneus. Similarly, negative contingencies with sad faces resulted in increased activation in medial frontal regions, extending into the superior frontal gyrus and motor-related areas during early trials. In contrast, participants’ ratings in positive contingency conditions remained consistent across trials, with similar behavioural and neural responses showing no significant differences between happy and sad face conditions. Our findings align with previous research addressing these anatomical regions involved in contingency learning (Tsukiura et al., 2003; Liljeholm et al., 2011; Morris et al., 2022; Saylik et al., 2022) and the processing of negative emotional stimuli (Bush et al., 2000; Kanske and Kotz, 2011; Pedale et al., 2019; Salsano et al., 2024). Moreover, our study provides further insights into how emotional stimuli modulate social interactions and judgment processes across contingency learning tasks.

The activation of the ACC and IFG is well-documented, highlighting their roles in conflict resolution, adaptive decision-making, the processing of negative emotional valence, and the handling of environmental dynamics like volatility and uncertainty (Aron et al., 2004; Aron, 2007; Behrens et al., 2007; Peters et al., 2017; Stolyarova et al., 2019; Klein-Flügge et al., 2022). The ACC, as part of a distributed network that includes lateral frontal areas, is particularly active during the modulation of causal perception in uncertain situations, such as zero contingency tasks and in response to negative emotional stimuli (Bryden et al., 2011; Palomero-Gallagher et al., 2019; Stolyarova et al., 2019). The ACC connects to lateral and medial frontal regions (e.g., IFG, SFG, MedFG), forming cognitive networks with subcortical areas (e.g., putamen, amygdala, hippocampus, and thalamus) anchored in the entorhinal cortex (Reznik et al., 2023; Syversen et al., 2024). This network, with the temporal gyrus acting as a hub, integrates and updates information from both cognitive and emotional domains (Krug and Carter, 2011; Spiers et al., 2017; Stolyarova et al., 2019; Horibe, 2024). In this context, regions such as the insula, which is involved in emotional salience, and the putamen, which is sensitive to the accumulation of various types of information, may play a crucial role in bridging the gap for making perceptual decisions in tasks where learning associations between stimuli is crucial (Behrens et al., 2007; Li et al., 2020; Salsano et al., 2024). Thus, the broader activation observed in the IFG and ACC, extending into temporal and subcortical regions, may reflect the increased cognitive demands of processing uncertain or ambiguous contingencies, particularly those involving negative emotional stimuli in zero contingency tasks.

Understanding the contingency-specific effects of emotional stimuli on causal perception requires examining both the characteristics of the emotional stimuli and the type of contingency involved. The altered perception of causality in zero contingency tasks with sad faces, compared to happy faces, likely arises from the uncertainty inherent in both sad faces and zero contingency conditions. Uncertain situations heighten stress and arousal, demanding significant cognitive resources related to attention and learning in order to resolve ambiguity (Peters et al., 2017). Zero contingency tasks, characterized by the absence of predictive relationships between cues and outcomes, represent a form of uncertainty that can induce stress and require substantial cognitive and neural resources to manage (Behrens et al., 2007; Matute et al., 2015, 2019; Msetfi et al., 2015; Stolyarova et al., 2019). This inherent ambiguity makes the judgment of zero contingency tasks particularly challenging, often triggering cognitive biases and mental shortcuts that hinder the accurate perception of cue-outcome relationships (Tiedens and Linton, 2001; Blanchette and Richards, 2010; Matute et al., 2015; Stolyarova et al., 2019).

Consistently, sad emotional stimuli are associated with uncertainty, negativity, and stress-related arousal (Tiedens and Linton, 2001; Calvo and Nummenmaa, 2008; Yuan et al., 2023). These features of negative emotional stimuli, such as sad faces, can trigger task-irrelevant mental activities that interfere with task-related processes, requiring greater effort during challenging cognitive tasks (Bush et al., 2000; Dolcos and McCarthy, 2006; Kanske and Kotz, 2011). In contrast, happy faces are the only positive expression among the six basic emotions, making them rewarding, pleasant, and uniquely distinguishable both in physical appearance and psychological impact when compared to other basic expressions (Calvo et al., 2018; Barros et al., 2023). Various studies highlight the pronounced salience of happy faces, demonstrating higher visual contrast in tasks like search and detection, speed-accuracy response systems, and machine learning analyses, which facilitate the allocation of attentional resources (Calvo et al., 2016, 2017, 2018; Calvo and Marrero, 2009; Calvo and Nummenmaa, 2008; Stuit et al., 2023). Consistent with our results, the distinct features of emotional expressions suggest that emotional content can lead to an overestimation or underestimation of the associative value between a cue and an outcome, particularly in zero-contingency situations (Averbeck and Duchaine, 2009; Evans et al., 2010).

We can speculate possible reasons why facial expressions differentially impact learning in zero contingency tasks, drawing on contingency learning and emotion-related theoretical frameworks. We have discussed how the characteristics of the faces and zero-contingency tasks might influence causal perception. One perspective might reflect on the previous predictability of different facial expressions interacting with saliency and attentional system. Mackintosh (1975) proposed a theory of selective attention, suggesting that cue validity depends on both physical salience and reliability of cues as predictors of outcomes. According to this theory, we focus on task-relevant cues while ignoring irrelevant ones, with more salient cues drawing attention and being learned more easily (Aisbitt and Murphy, 2016). This idea aligns with the attention-narrowing hypothesis, which suggests that negative emotions interfere with cognitive processes by heightening arousal (which leads to task-irrelevant mental activity) and narrowing attention to specific details during decision-making (Easterbrook, 1959; Wichary et al., 2016; Saylik, 2017).

An alternative view suggests that perception of causality may be driven by predictive value than actual value in zero contingency situations, particularly when affective features are involved (Yarritu and Matute, 2015; Matute et al., 2019). As a result, individuals are more likely to perceive connections between unrelated events based on emotions, prior beliefs, expectations, mood, or emotional cues (Thornton and Tamir, 2017; Anderson et al., 2019; Matute et al., 2019; Barrick et al., 2024). This aligns with the availability heuristic hypothesis, which proposes that emotions shape judgments by enhancing access to mood-congruent information (Blanchette and Richards, 2010). Recent research supports this idea, showing that participants are more likely to judge zero-contingency tasks negatively when exposed to undesirable stimuli, such as sad faces. In contrast, desirable stimuli, like happy faces, lead participants to overestimate outcomes and provide more positive ratings (Fulcher et al., 2001).

In summary, applying this to our findings, pairing sad stimuli with zero-contingency tasks may increase uncertainty and stress-related arousal, requiring more cognitive resources and strategies, which in turn increases activity in cognitive control areas. In contrast, happy expressions-associated with approachability and stronger saliency-may serve as more reliable predictors of causal outcomes, thereby facilitating associative processes.

Although negative contingency generally did not show significant behavioural or neural differences across the stimuli manipulation, an analysis of the early trials revealed that sad faces elicited a weaker perception of causality compared to happy faces. This was accompanied by increased activity in frontal areas (MFG, SFG and ACC), which was similar to the activation patterns observed in zero-contingency tasks. It appears that forming predictions was easier when contingencies were paired with happy faces, but more demanding when paired with sad faces, particularly during the early trials. One explanation for this is that forming predictions in negative contingencies is more challenging than positive ones. This is because it is harder to detect a cue signal when it is not followed by an outcome, and vice versa. Therefore, the observed divergence in early trials between sad and happy faces is likely not due to uncertainty effects in zero-contingency tasks but rather the increased cognitive load associated with processing negative emotional stimuli, combined with the challenging nature of negative contingencies (Maldonado et al., 2006; Heisz et al., 2011; Saylik et al., 2022). The diminished effect observed in the late trials seems to be related to increased certainty, as reliable information accumulates over time. This suggests an adaptation or learning process, where participants adjust their judgments as they gather more information (Mackintosh, 1975; Behrens et al., 2007; Murphy and Castiello, 2024). Therefore, the divergence in the early trials and the convergence in the late trials indicates that inferring cue-outcome relationship is based on the total accumulated information (Mackintosh, 1975; Behrens et al., 2007). Thus, the biased perception in happy and sad face conditions reduces after experiencing sufficient number of trials (Murphy et al., 2011). That is to say, in the early trials of a contingency participants are still forming their judgment along with limited information to infer cue-outcome relationships while sad faces may interfere and happy faces facilitate the process, however as the trials progresses to the end, they receive more information and adjust their judgments (Murphy et al., 2011).

Finally, our findings integrate well with theoretical models of contingency learning and emotional processing. The Delta P (∆P) rule, as introduced by Allan (1980), provides a robust framework for understanding how individuals learn and discriminate between different types of contingencies. Our results extend this model by incorporating the influence of emotional valence, demonstrating that emotional stimuli engage distinct neural circuits and modulate the learning and evaluation of contingencies.

The current study has several limitations that should be addressed in future research. First, we used fixed Delta *p* values (0.50, 0, −0.50) with a limited number of trials and ratings (16 trials per condition with 8 repetitions/ratings) and only two emotional expressions (happy and sad). Previous research indicates that biased perceptions of causality tend to disappear after experiencing a sufficient number of trials, with early biases diminishing over time (Murphy et al., 2011; Matute et al., 2019). While we observed this effect in the negative contingency, the divergence persisted in zero-contingency tasks through stimuli manipulation. Second, although we applied some exclusion criteria (e.g., participants’ age, gender, and psychiatric history), certain individual differences, such as alexithymia, were not considered. Future studies should explore varied contingency settings (e.g., longer trials, increased ratings per condition) and a broader range of emotional expressions (e.g., anger, fear) while examining the long-term effects of emotional contingencies on social interactions and decision-making, taking individual traits like alexithymia and neuroticism into account (Saylik et al., 2018; Cuve et al., 2021). Third, in our analysis, we applied an FHWE correction threshold of 0.05 but when this is not significant, we examined uncorrected significant thresholds at the cluster level (e.g., uncorrected at 0.001). Particularly, for the analysis of the first and second trials, we adopted more liberal thresholds due to the reduced number of trials. Future research should take this into account when interpreting our findings.

In conclusion, the findings suggest that sad stimuli heighten cognitive demands in tasks with zero or partially negative contingencies eliciting weaker perception of causality and activating contingency-related brain areas more than happy stimuli. This underscores the intricate relationship between emotional processing and causal learning, with facial expressions influencing causal perception, potentially overriding more rational judgments (Furl et al., 2012). The results suggest that certain emotional experiences have a stronger influence on shaping the judgments. Our findings integrate well with theoretical models of contingency learning and emotional processing. The Delta P (∆P) rule, as introduced by Allan (1980), provides a robust framework for understanding how individuals learn and discriminate between different types of contingencies. Our results extend this model by incorporating the influence of emotional valence, demonstrating that emotional stimuli engage distinct neural circuits and modulate the learning and evaluation of contingencies.

## Data Availability

The datasets presented in this study can be found in online repositories. The names of the repository/repositories and accession number(s) can be found below: 10.6084/m9.figshare.27651537.
